# Distribution Patterns of *Platycodon grandiflorus* From the Last Interglacial Period to the Future by Ecological Niche Modeling

**DOI:** 10.1002/ece3.71198

**Published:** 2025-03-30

**Authors:** Chun‐Jiao Li, Xin‐Tong Xie, Tuo Shi

**Affiliations:** ^1^ College of Life Science, Shenyang Normal University Shenyang Liaoning China

**Keywords:** climate change, conservation strategies, MaxEnt model, *Platycodon grandiflorus*, potential distribution

## Abstract

Global climate change may represent a significant threat to the distribution and quality of medicinal plants, altering cultivation areas and compromising the quality of medical materials. *Platycodon grandiflorus* , a traditional Chinese medicinal herb, has a millennia‐long medicinal and culinary use history in East Asia. Given its escalating demand, accurately evaluating the changes under different climate scenarios and predicting its potential distribution are imperative for ensuring its conservation and sustainable utilization. By integrating MaxEnt with ArcGIS, this study advances previous approaches by incorporating historical, present, and future climate data to model the distribution dynamics of *P. grandiflorus* across China. The results indicated: (1) The species' distribution strongly correlates with environmental variables, particularly bio13, prec07, prec09, and tmin07, whose cumulative value of percent contribution was 78.5%; (2) The centroids of potential geographic distribution during the LIG, LGM, and MH periods were situated further westward compared to the present distribution, with substantial contraction observed in highly suitable habitats throughout these historical periods; (3) Under present climatic conditions, the overall suitable habitat encompasses 4,185,964 km^2^, with highly suitable habitats constituting one‐third of this expanse, predominantly concentrated in central, southern, and northeastern China; (4) Future climate change scenarios predict that the total suitable habitat will expand to varying degrees (7% increase on average), albeit with potential reductions in highly suitable areas (3% decrease on average); and (5) The distribution of *P. grandiflorus* is likely to move toward higher latitudes in the future due to climate changes. Our findings fill a critical knowledge gap by quantifying the impact of climate change on the distribution of *P. grandiflorus* . These results offer crucial insights for developing effective conservation strategies, promoting sustainable utilization, and establishing standardized cultivation protocols for *P. grandiflorus* resources.

## Graphical abstract

Global climate change might lead to alternations in cultivation areas and the quality of medical materials since it causes changes in suitable areas for growing medicinal plants. *P. grandiflorus* , a traditional Chinese medicine, has medicinal and culinary value for thousands of years in East Asia. With its increasing demand, predicting the impact of climate change on the distribution of *P. grandiflorus* is crucial for its protection and the sustainable use of resources.

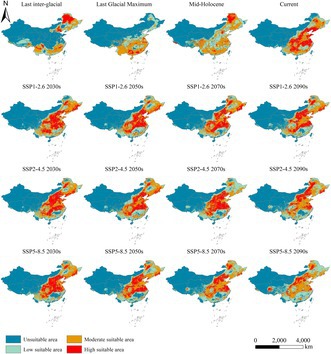

## 1 Introduction

The impact of climate on organisms is widespread and far‐reaching, and it is a globally significant issue (Jump and Peñuelas 2005). Climate changes influence surface temperature, precipitation, heatwave frequency, forest fires, and pest outbreaks (Gao et al. 2024). Collectively, these variables (and their interactions) modify plant adaptability pressure, community composition, and ecosystem structure (Almeida et al. 2023; Wang et al. 2023; Qin et al. 2017; Thuiller et al. 2011; Pio et al. 2014), thereby changing geographic distribution patterns of plants. Such changes may lead to large‐scale expansion or extinction of species geographic distribution (Zhang et al. 2018), undoubtedly bringing severe challenges to biodiversity conservation (Cursach et al. 2018). Thus, explaining the patterns of interaction between plants and climate dynamics is an important challenge for contemporary research (González‐Orozco et al. 2016; Bellard et al. 2012).

Numerous research suggests that changes in the global climate will alter the distribution of suitable plant habitats (Bellard et al. 2012; Kaky and Gilbert 2017). Quaternary climatic oscillations between glacial and interglacial stages have significantly influenced the current patterns of plant geographical distribution (Hewitt 2004). During these oscillations—the last interglacial (LIG, 120 kyr), the last glacial maximum (LGM, 21 kyr), and the mid‐holocene (MH, 6 kyr), the LIG period is similar to the contemporary climate (Fačkovcová et al. 2017). However, the LGM is one of the Earth's extreme periods of environmental changes (Clark et al. 2009). The extreme cooling during the LGM led to some species' extinction, while many surviving species were forced into glacial refugia, dramatically reducing the suitable habitats (Nogués‐Bravo et al. 2010). After the postglacial warming, surviving populations began refugial expansion and recolonized areas (Davis and Shaw 2001; Normand et al. 2011; Sworobowicz et al. 2020; Zani et al. 2023). Nevertheless, the past influence of climate change and intrinsic biological limitations shape modern biogeography (De et al. 2021). Species dispersal ability, reproductive rates, and habitat specificity may impede the process of recolonization, resulting in the emergence of endemic and disjunctive distributions that are observed at present (Sandel et al. 2011; Svenning and Skov 2007; Feng et al. 2019; Coello et al. 2024). These patterns may reflect the historical climate influence on modern species distributions. These past dynamics can then be rebuilt to understand better how species have determinedly adjusted to a warming climate in the past (Blois et al. 2013). Most importantly, we could predict how species react to future change across various aspects, including species range, extinction risk, and population dynamics (Forester et al. 2022). Knowledge of these mechanisms can enable us to anticipate future challenges of climate change and conservation efforts to protect endangered species and ecosystems (Bai et al. 2018; Provan and Bennett 2008).

Ecological niche modeling has recently been used to investigate responses of geographical ranges of plant species to climate change. The niche models are based on the niche theory and estimation algorithms (Zhao et al. 2021). Plant niche characteristics are discovered by exploring the geographic distribution and the corresponding climate data that enable the prediction of appropriate areas in which the species will thrive based on the explored climatic conditions in space and time, to with past, present, and future (Kearney et al. 2010; Peterson et al. 2011; Zhang et al. 2019). Some of the most frequently used models today are the maximum entropy model (MaxEnt) (Phillips et al. 2006), the maximum likelihood‐based method (MaxLike) (Phillips and Elith 2013; Merow and Silander Jr. 2014), the generalized additive model (GAM) (Hastie 2017), and BIOCLIM and HABITAT model (Leathwick et al. 1988; Latimer et al. 2006). Among all predictors, MaxEnt is considered one of the best and is commonly used with large presence‐only datasets or incomplete datasets (Qiao et al. 2015; Gao et al. 2020). It is also important to emphasize that the potential distribution of plants we simulated is generated by the MaxEnt niche model, which has the characteristics of lower distortion and higher stability (Zhang et al. 2021). This approach identifies regions meeting a species' niche requirements, predicting where it may potentially occur (Martínez‐Meyer et al. 2013).

The most common species, *P. grandiflorus* , is perennial and a member of the bellflower family (Campanulaceae) and is found in some Asian countries, including China, Korea, Japan, and Eastern Siberia (Lammers 2007a, 2007b). *P. grandiflorus* is a disjunctive species in China, distributed in the North, South, and east regions except for the Qinghai‐Tibet region (Hong et al. 2011). This species has been used in traditional Chinese medicine for millennia because of its potent pharmacological effects. It is rich in bioactive elements, such as saponins, flavonoids, phenolic acids, and polyacetylene (Wei 2005; Li, Wang, et al. 2016; Sui et al. 2014; Kim et al. 2020), which have anti‐inflammatory effects, anti‐obesity activity, anti‐cancer properties, and anti‐allergy effects (Li and Yang 2021; Ke et al. 2020; Buchwald et al. 2020; Wang et al. 2017; Nyakudya et al. 2014; Zhang et al. 2015, 2022; Lee et al. 2020). Furthermore, its roots are rich in proteins, iron, calcium, trace elements, vitamins, and essential amino acids, which could be used for dietary supplements (Kumar et al. 2017; Marles 2017). Moreover, the flowers of *P. grandiflorus* are in the shape of a hanging clock and are blue, purple, and white, making it one of the most ornamental and popular flowers (Wang et al. 2018; Ji et al. 2020; Lv et al. 2021).

Global climate change has significant effects on the different aspects of medicinal plants. For example, the daily temperature difference and precipitation in the coldest season can influence the quality of medicinal materials by regulating key genes related to the alkaloid biosynthesis pathway in some medicinal plants (Li and Yang 2021). Polysaccharides, total alkaloids, and total flavonoids in cultivated *Dendrobium* officinale were influenced by 16 ecological factors, such as humidity, sunshine duration, soil pH, and soil total nitrogen (Yuan et al. 2020). The maximum temperature of the warmest month and precipitation of the warmest quarter are the two that most determine the geographical distribution of a medically used plant, *Fritillaria cirrhosa* (Zhao et al. 2018). Similar large‐scale studies have been performed on traditional Chinese medicinal plants from LIG to the present (Du et al. 2021; Li et al. 2020; Wei et al. 2018; Xu et al. 2022).

*P. grandiflorus* has long been used as a medicinal and edible plant, with significant market demand. Thus, the sustainable development of *P. grandiflorus* is essential. Due to overharvesting and depletion of wild resources, *P. grandiflorus* must now be grown in large‐scale cultivation. Selecting suitable planting regions is pivotal for the industrialization of *P. grandiflorus* . However, most research on *P. grandiflorus* has primarily focused on chemical constituents and pharmacological activities (Zhang et al. 2015, 2022; Ma et al. 2024; Dong et al. 2021; Zou et al. 2021). Therefore, few analyses have considered how suitable habitats of *P. grandiflorus* respond to global climate change (Dong et al. 2019). Using MaxEnt modeling with ArcGIS, we predicted the suitable habitats of *P. grandiflorus* under past, present, and future climatic conditions. We also evaluated the dominant environmental drivers of changes in its distribution patterns. In addition, modeling past distribution dynamics of *P. grandiflorus* allows us to understand better the process that determined its formation and changes in distribution by region. Moreover, the identification of migration routes based on previous distribution patterns enables the definition of protective measures for vegetation under climate change, which can be applied in the future (Werneck et al. 2011; Yu et al. 2017). The aim of this study was to (1) reconstruct the geographical distribution patterns of *P. grandiflorus* since the LIG time, (2) assess the environmental variables influencing its potentially suitable range, and (3) supply a scientific foundation for the protection and management of *P. grandiflorus* . Our data is important for determining appropriate habitats and the sustainable use and conversion of *P. grandiflorus* .

## 2 Materials and Methods

### 2.1 Species Occurrence Records

**FIGURE 1:**
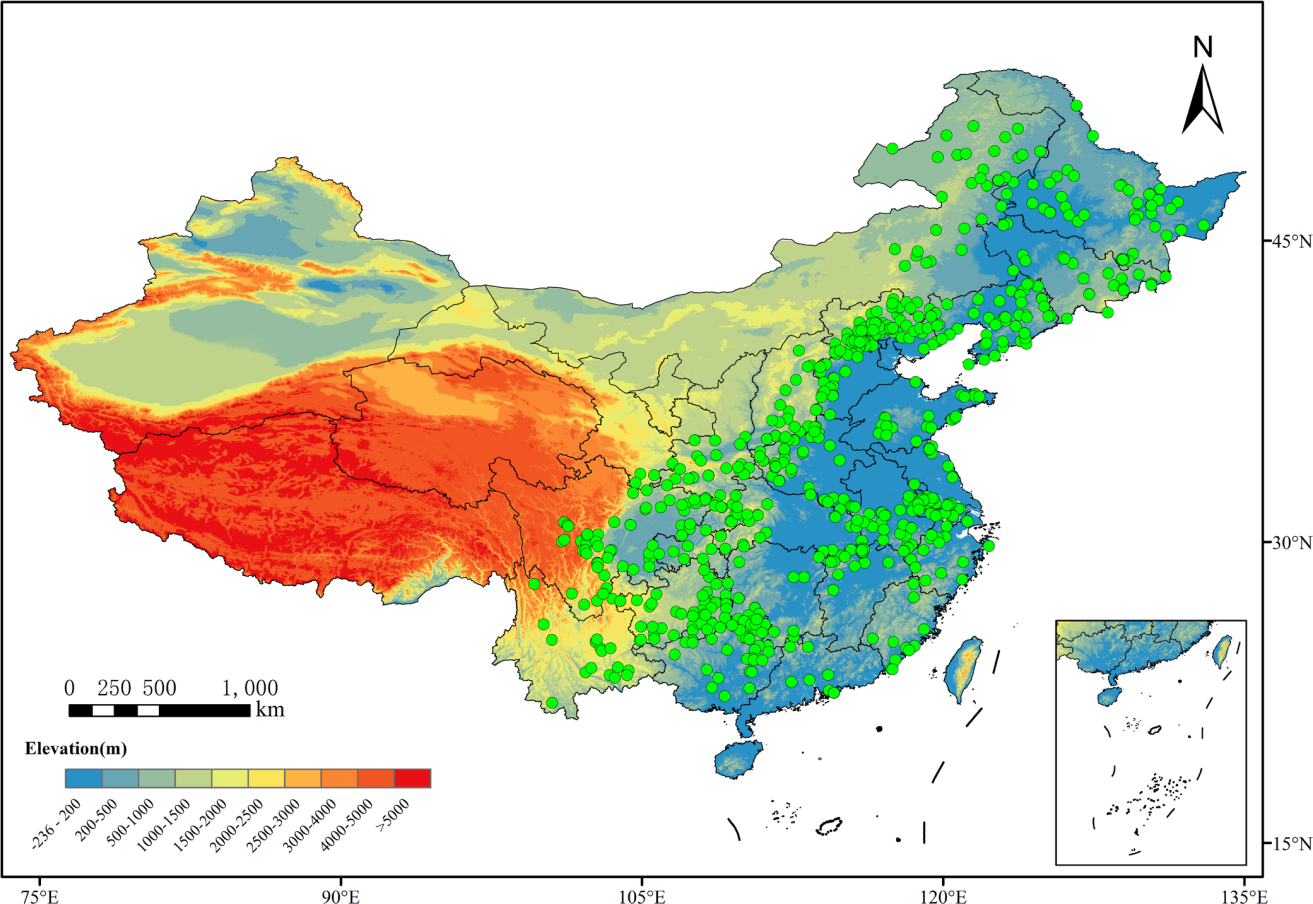
Distribution of the sample points of *P. grandiflorus* in China.

The geographical distributions of *P. grandifloras* in China were primarily generated from the Global Biodiversity Information Facility (GBIF, https://www.gbif.org/), supplemented by data from the Chinese Virtual Herbarium (CVH; available at http://www.cvh.ac.cn) and the National Specimen Information Infrastructure (NSII; available at http://www.nsii.org.cn). We deleted all locations without detailed latitude and longitude. For records with specific location descriptions, accurate geographic coordinates were obtained using Google Earth (https://earth.google.com/), yielding 1688 geographical distribution records for *P. grandifloras* (Figure 1). Subsequent data refinement involved the removal of erroneous and duplicate records. A grid with 30″ × 30″ cells was established utilizing QGIS v3.28.4. For each cell, only the distribution point nearest to the centroid was retained (Merow et al. 2013). The processed data was then converted into CSV format for further analysis.

### 2.2 Environmental Data Acquisition, Screening, and Processing

This study utilized 55 environmental variables, encompassing 36 climatic and 19 bioclimatic factors (Table 1), derived from the WorldClim Database (https://worldclim.org). These variables cover multiple periods, including LIG, LGM, Mid‐Holocene, recent historical period, and future predictions for the 2030s (2021 to 2040), 2050s (2041 to 2060), 2070s (2061 to 2080), and 2090s (2081 to 2100). Preprocessing was conducted using ArcGIS v10.8.2 software to ensure data consistency (Ayob et al. 2023). This process involved removing invalid data, filling in missing values, and standardizing the spatial resolution to 30″(approximately 1 km^2^) (He et al. 2023). All variables were projected to the WGS 1984 coordinate system (World Geodetic System, 1984).

**TABLE 1:** Climate variables used to build models.

Environmental factors	Meaning	Unit
Bio1	Annual mean temperature	°C
Bio2	Mean diurnal range	°C
Bio3	Isothermality	—
Bio4	Temperature seasonality	—
Bio5	Max temperature of warmest month	°C
Bio6	Min temperature of coldest month	°C
Bio7	Temperature annual range	°C
Bio8	Mean temperature of wettest quarter	°C
Bio9	Mean temperature of driest quarter	°C
Bio10	Mean temperature of warmest quarter	°C
Bio11	Mean temperature of coldest quarter	°C
Bio12	Annual precipitation	mm
Bio13	Precipitation of wettest month	mm
Bio14	Precipitation of driest month	mm
Bio15	Precipitation seasonality	—
Bio16	Precipitation of wettest quarter	mm
Bio17	Precipitation of driest quarter	mm
Bio18	Precipitation of warmest quarter	mm
Bio19	Precipitation of coldest quarter	mm
Tmin0 ~ 12	Monthly minimum temperature	°C
Tmax0 ~ 12	Monthly maximum temperature	°C
Prec0 ~ 12	Monthly precipitation	mm

The baseline for the initial model was current climate data (1970–2000). CMCC‐ESM2: the second‐generation CMCC Earth System Model, which covers more biogeochemical processes than the CMIP5 framework. This sparsely capturing model has been validated correctly with historical simulations and high‐quality performance (Lovato et al. 2022). Climate data predicted for future scenarios were retrieved from the CMCC‐ESM2 model from the WorldClim database (although this model was not the excursion from WorldClim, the nearest in the cases presented below) to make the different periods comparable. Some recent works adopted the coupled model intercomparison project phase 6 (CMIP6) based on shared socioeconomic pathways (SSPs) to evaluate the outcomes of global climate change over the presumable distributions of *P. grandifloras*, adding three future climate scenarios for our research, SSP1‐2.6 (the sustainable pathway), SSP2‐4.5 (the medium pathway), and SSP5‐8.5 (fossil‐fueled development) (Ai et al. 2024). These recent uses of these scenarios can be identified in habitat suitability studies (Shi et al. 2024; Zhang et al. 2024). SSP1‐2.6 integrates SSP1 and RCP2.6, representing a sustainable future with a low greenhouse gas emissions scenario. SSP2‐4.5 combines SSP2 and RCP4.5 and describes a moderate socioeconomic development scenario with intermediate emissions levels. In contrast, SSP5‐8.5 merges SSP5 and RCP8.5, reflecting rapid economic growth fueled by heavy fossil energy consumption and resulting in high carbon emissions (O'Neill et al. 2016, 2017).

On the contrary, collinearity among environmental variables often leads to model overfitting, which diminishes predictive performance (Araújo et al. 2019; Miller 2010; Miller et al. 2020; Bradie and Leung 2017). A systematic variable screening method was performed to ensure that training models were performed as accurately as possible. We removed variables explaining < 0.5 due to their performance in the MaxEnt model. We used the software package to perform statistical analyses (SPSS version 18). For variable pairs with |*r*| > 0.8, only the variable contributing most toward the model was retained (Dormann et al. 2013; Zuur et al. 2010; Zhao et al. 2021; Fang et al. 2024). We also considered the Jackknife test output when selecting highly predictive variables with large relevance values for modeling.

### 2.3 Construction, Optimization, and Evaluation of MaxEnt Model

MaxEnt is mainly used for species distribution modeling with presence data (Elith et al. 2011). Feature combination (FC) and regularization multiplier (RM) are the main parameters influencing MaxEnt model performance (Phillips and Dudík 2008). MaxEnt implements features using five methods: linear (L), quadratic (Q), product (P), threshold (T), and hinge (H) (Hernández‐Lambraño et al. 2021). The FC option default is derived from the sample variability using an RM value of 1 (Elith et al. 2011). However, It has been demonstrated that default settings available on MaxEnt tend to overfit (Elith and Graham 2009).

MaxEnt model parameters were optimized in the R package ENMeval by changing FC and RM. This minimizes the model space, leading to better predictive performance and transferability of the currently developed model (Muscarella et al. 2014; Kass et al. 2021). Six different FC combinations were examined: L, LQ, H, LQH, LQHP, and LQHPT. We estimated 78 combinations of parameters from 13 values of the regularization multiplier from 0.1 to 4 by step of 0.5. The best‐fit parameter combination is ultimately determined using the corrected Akaike Information Criterion (AICc), which balances model accuracy and complexity. The best model was selected with the lowest delta AICc value (Warren and Seifert 2011). The best fit has been evaluated RM = 1.0 and FC = LQHPT (deltaAICc = 0).

MaxEnt software (v3.4.1) was performed to forecast the potential distributions of *P. grandifloras* (https://biodiversityinformatics.amnh.org/open_source/maxent/). Environmental variables were chosen and processed with the distribution data to import into MaxEnt. We applied the best parameter settings based on the model optimization results and enabled the ‘Create response curves’ option. The relative importance of the environmental factors was examined using the simplex method. The output type was set to Logistic, a more flexible fit (Phillips and Dudík 2008), and the replicated run type to Bootstrap. The distribution data were separated into 75% for training and 25% for testing (Li et al. 2024). The model was run 10 times, and the mean values from all runs were selected as the ultimate output to minimize errors. We set the ‘Randomseed’ option to give a better model's randomness. Of the data, the data block was key for analysis and was preserved for additional analysis and visualization. Other parameters were used without modification from their default configurations.

The mean of ten model runs was imported into ArcGIS v10.8.2 to simulate spatial visualization (Shi et al. 2024). According to the Jenks natural breaks classification, to aid in separating the predicted distribution probability, four thresholds were created: unsuitable habitats (less than 0.11), lowly suitable habitats (0.11 to 0.30), moderately suitable habitats (0.30 to 0.47), and highly suitable habitats (more than 0.47). Such classification delineated suitable niches of *P. grandifloras* in China (Guga et al. 2021; Ma et al. 2021). The area of these suitability classes was calculated for all climate scenarios using ArcGIS. The SDMToolbox in ArcGIS (http://www.sdmtoolbox.org/) was used to calculate the centroid (geometric center) of suitable habitats across scenarios. The predicted routes of historical migrations were evaluated, and the predicted centroid of suitable habitats was examined for the future (Brown 2014; Zurell et al. 2020).

The accuracy of the MaxEnt model predictions was evaluated using the receiver operating characteristic curve (AUC) (Yan et al. 2020; Zhang et al. 2021). AUC values closer to 1 indicate a more predictive model. AUC values larger than 0.9 demonstrated excellent predictive performance, values of 0.8 to 0.9 showed good exhibition, and values < 0.8 were suggestive of fair, poor, or inaccurate predictions (Wang et al. 2007).

## 3 Results

### 3.1 Precision and Dominant Environmental Variables

**FIGURE 2:**
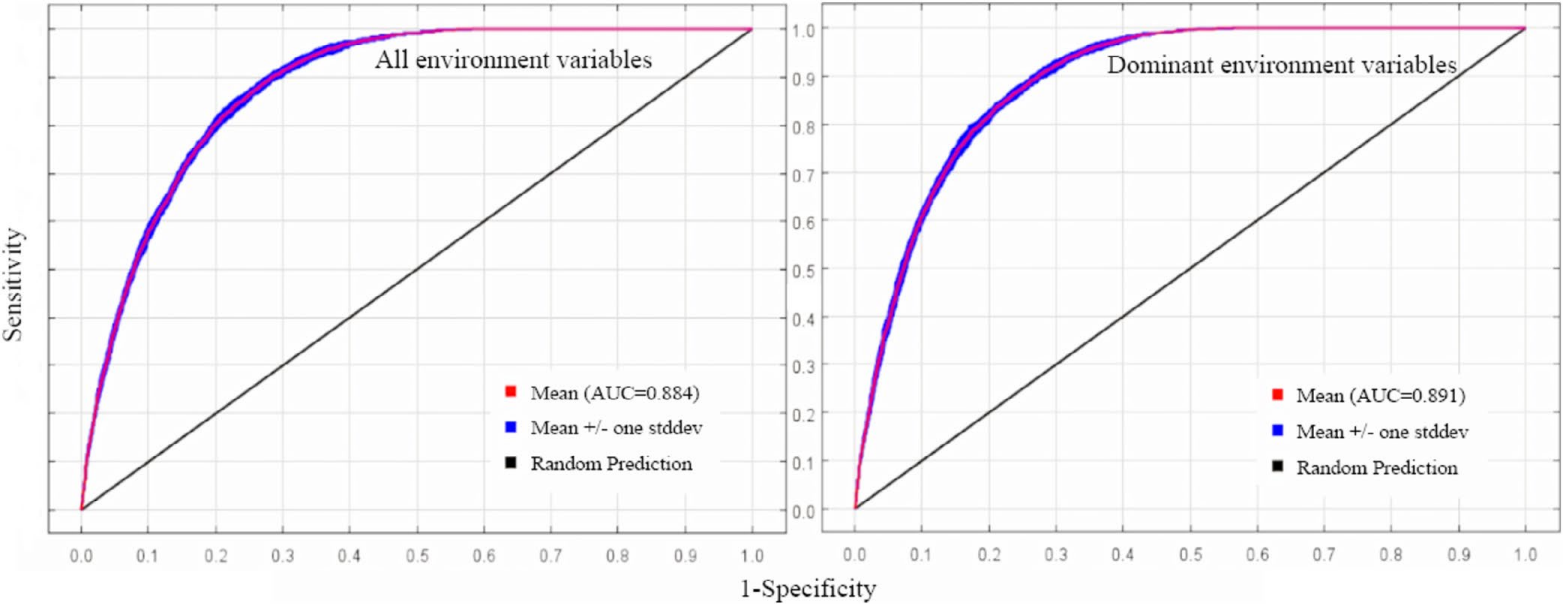
Receiver operating characteristic curve of potential distribution under all and dominant environmental variables.

Using 761 screened distribution sites and 11 climate variables (Table 2; Figure S1), the MaxEnt model was constructed to simulate the potentially suitable habitats for *P. grandiflorus* . The model's predictive accuracy was estimated based on the AUC value. The MaxEnt model displayed an average AUC value of 0.891 for dominant environmental variables (Figure 2), with a standard deviation of 0.0093. This demonstrated that the model reliably predicts the potential distribution of *P. grandiflorus* with high accuracy. Furthermore, the average AUC value for predictions using dominant variables was higher than that of models using all variables (0.884) (Figure 2). Therefore, using significant environmental variables effectively performed the potentially suitable habitats of *P. grandiflorus* with highly accurate results. This approach avoided the issue of overfitting (Swets 1988).

**TABLE 2:** Percent contribution and permutation importance of dominant climate variables.

Number	Variable	Percent contribution (%)	Permutation importance (%)
1	bio13	30.9	3.5
2	prec07 (July precipitation)	26	39.2
3	tmin07 (July minimum temperature)	17.1	12.9
4	tmax04 (April maximum temperature)	5.6	7.3
5	bio4	4.8	8.1
6	prec09 (September precipitation)	4.5	8.4
7	prec10 (October precipitation)	3	7.8
8	bio3	2.8	2.4
9	tmax06 (June maximum temperature)	2.5	2.6
10	prec01 (January precipitation)	1.9	5.1
11	bio2	0.8	2.6

**FIGURE 3:**
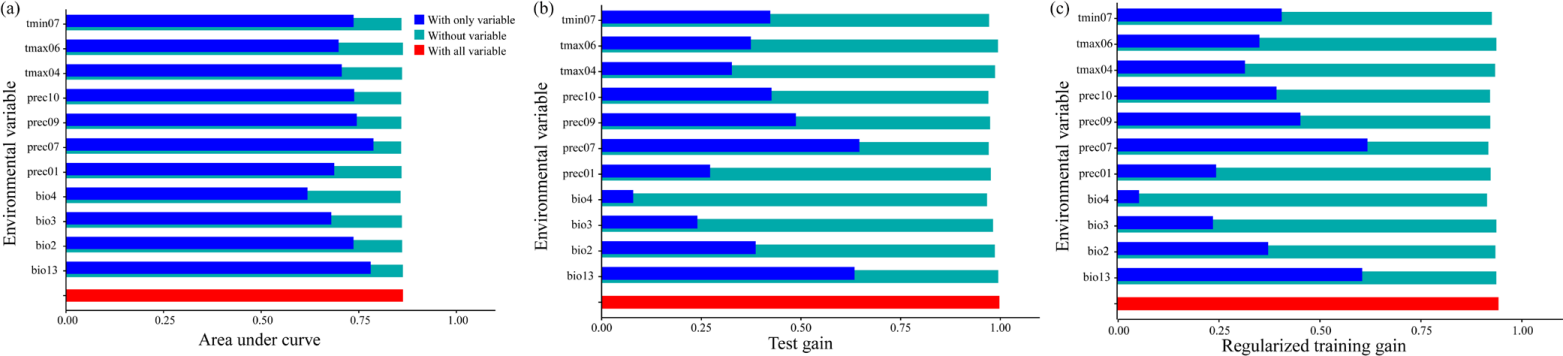
Jacknife test of the importance of variables. Blue, green, and red bars represent running the MaxEnt with the variable alone, without the variable, and with all variables (a) regularization training gain; (b) test gain; (c) AUC.

Among the eleven climate variables analyzed, precipitation of the wettest month (bio 13, 30.9% of variation), July Precipitation (prec07, 26% of variation), and July minimum temperature (tmin07, 17.1 of variation) contributed significantly more than other variables. Their cumulative contributions were as high as 74% (Table 2). The top three significant factors were prec07, tmin07, and September precipitation (prec09), accounting for a cumulative contribution of 60.5% (Table 2). The MaxEnt jackknife tests showed that bio13, prec07, and tmin07 achieved the maximum normalized AUC values. These results were consistent across regularized training gain, AUC, and test gain (Figure 3). Accordingly, the dominant factors shaping the contemporary geographical distribution of *P. grandiflorus* are precipitation‐related variables (bio13, prec07, and prec09) and temperature‐related variables (tmin07).

**FIGURE 4:**
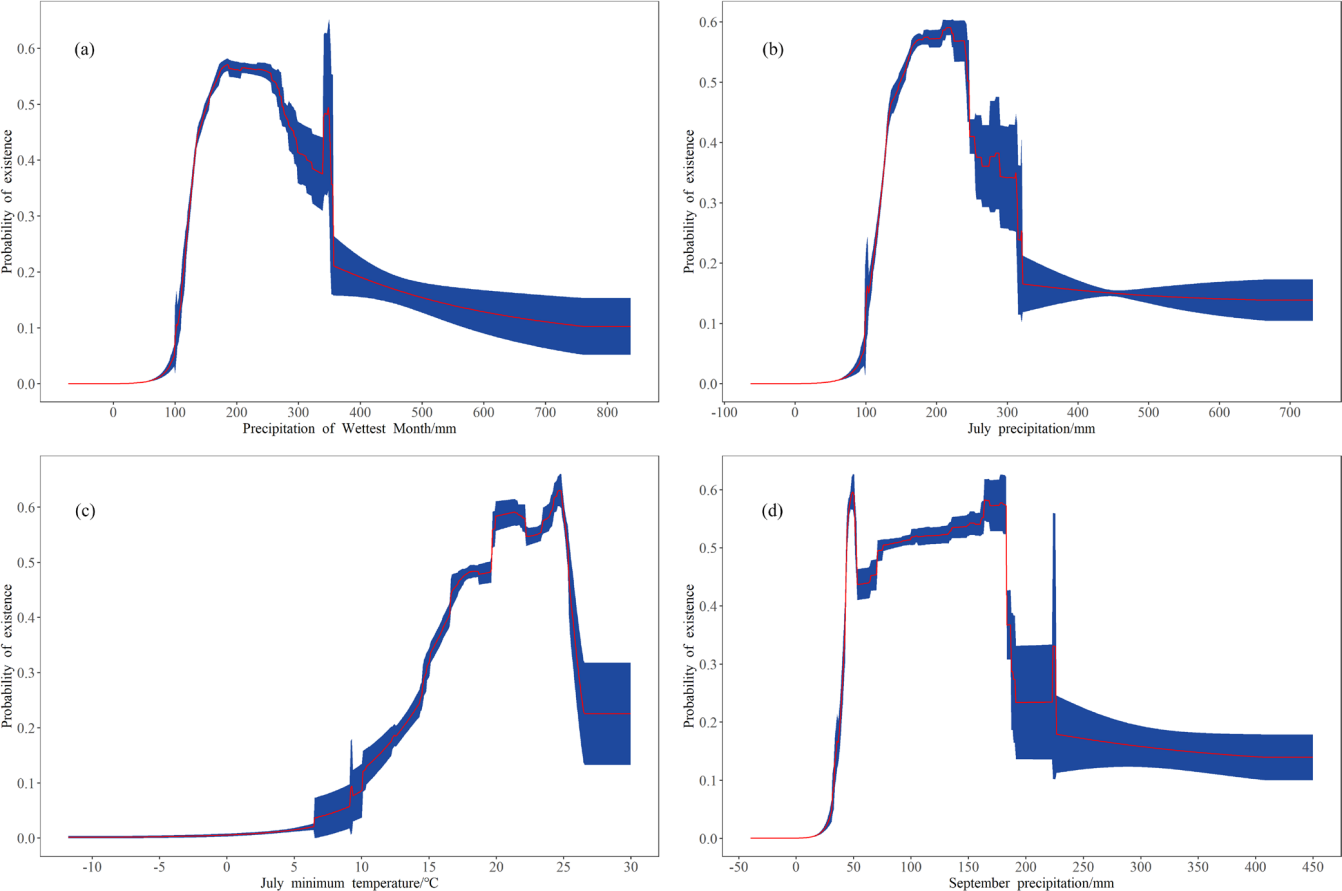
Response curves of the four most important environmental predictors in modeling habitat distribution for *P. grandiflorus* (a) Precipitation of wettest month (bio13)/mm; (b) July precipitation (prec07)/mm; (c) July minimum temperature (tmin07)°C; (d) September precipitation (prec09)/mm.

Figure 4 illustrates the response curves of major climate factors that influence the distribution of *P. grandiflorus* . The optimal range for precipitation of the wettest month (bio 13) was 154.8–271.3 mm, significantly affecting the species' occurrence (Figure 4a). The probability of occurrence was highest for July precipitation (prec07), with the range of 148.3–245.3 mm (Figure 4b). *P. grandiflorus* is preferred for relatively high July minimum temperatures (tmin07), with the optimum range of 19.7°C–25.3°C (Figure 4c). September Precipitation (prec09) showed higher probabilities between 44.4 and 182.4 mm (Figure 4d).

### 3.2 Historical Prediction

**FIGURE 5:**
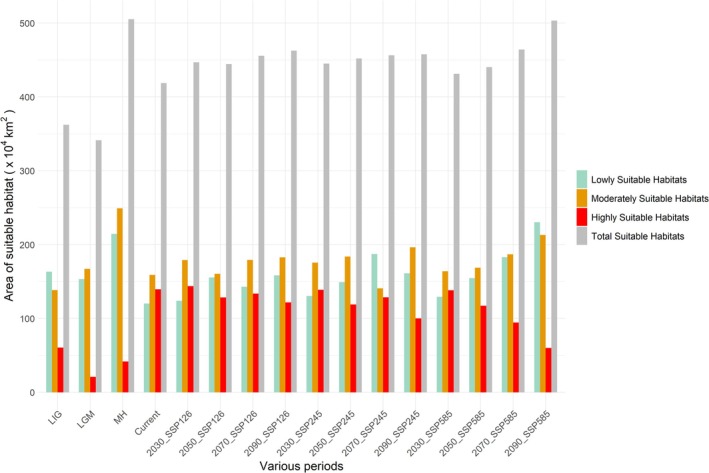
Area (×10^4^ km^2^) of suitable habitats for *P. grandiflorus* in China in various periods.

**FIGURE 6:**
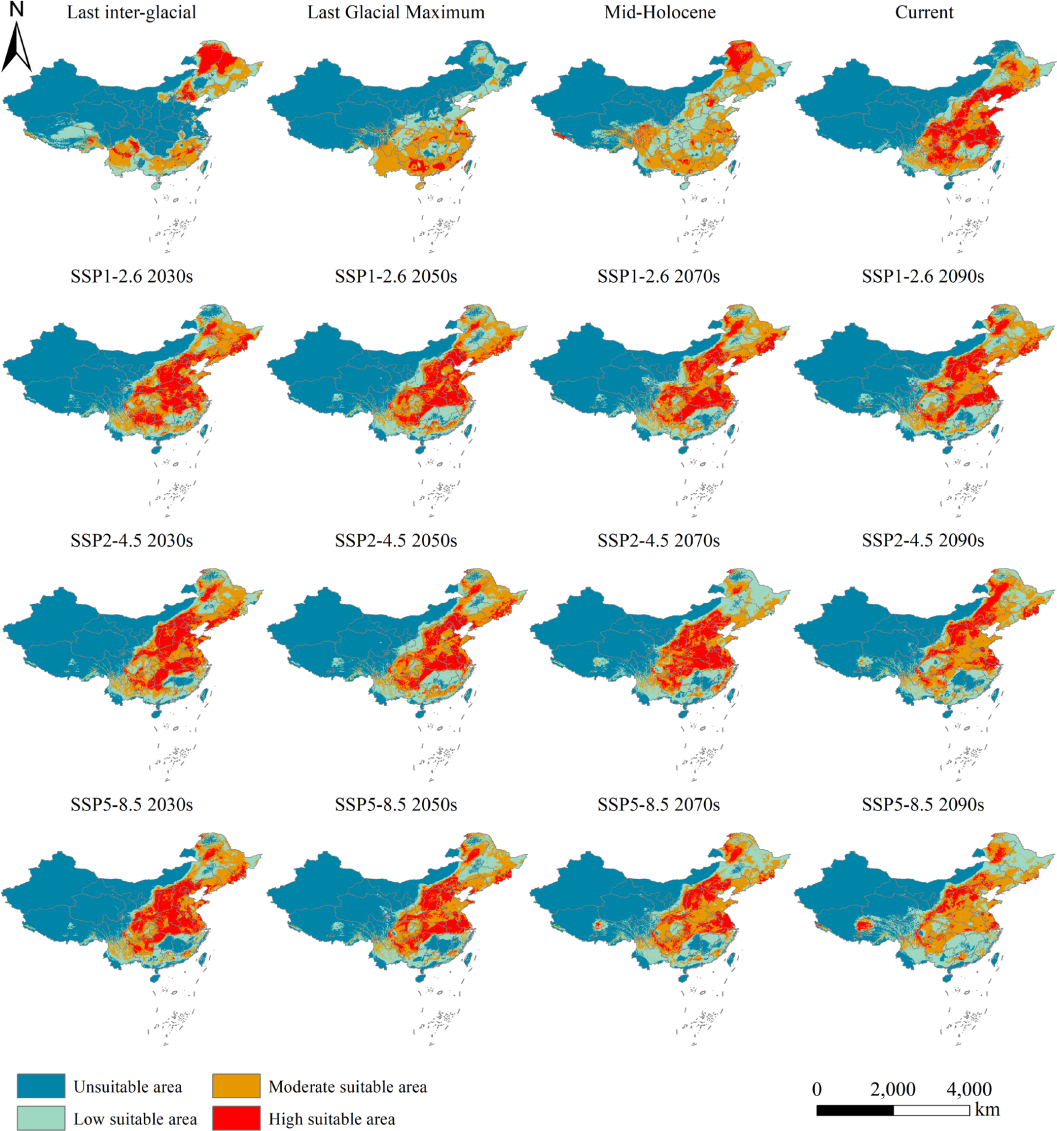
Distribution of current, historical, and future period habitats for *P. grandiflorus* in China.

The distribution of *P. grandiflorus* across different historical periods is depicted in Figures 5 and 6, and Table 3. During the LIG period, the suitable habitats for *P. grandiflorus* were restricted. Highly suitable habitats were in northeastern Inner Mongolia, northwestern Heilongjiang, Beijing, and eastern Hebei (Figure 6). The total area of suitable habitats was approximately 3,621,922.5 km^2^. Compared to other historical periods, the area of moderately suitable habitats was smaller, covering 1,384,050 km^2^. In contrast, highly suitable habitats expanded significantly, reaching 605,903.5 km^2^.

**TABLE 3:** Suitable habitat of *Platycodon grandiflorum* under different climate scenarios.

Climate scenario	Predicted area(×10^4^ km^2^)				
	Lowly suitable habitat	Moderately suitable habitat	Highly suitable habitat	Total suitable habitat	Unsuitable habitat
Last interglacial period	163.1969	138.405	60.59035	362.19225	598.8169
Last glacial maximum	153.1893	167.1634	20.92646	341.27916	620.6028
Mid‐holocene	214.3966	249.0569	41.71826	505.17176	455.8374
Current	120.1347	159.0112	139.4505	418.5964	543.2857
SSP1‐2.6					
2030s	123.9378	178.9953	143.7998	446.7329	515.1234
2050s	155.5425	160.3716	128.4952	444.4093	517.4702
2070s	142.903	179.1405	133.6133	455.6568	506.2251
2090s	158.1713	182.6415	121.7766	462.5894	499.2912
SSP2‐4.5					
2030s	130.4685	175.6444	138.8994	445.0123	516.8691
2050s	149.0944	183.8766	118.9379	451.9089	509.9567
2070s	187.142	140.6494	128.5697	456.3611	505.5038
2090s	161.1662	196.3425	100.1214	457.6301	504.252
SSP5‐8.5					
2030s	129.2462	163.9623	138.1087	431.3172	530.5641
2050s	154.5469	168.5999	117.0949	440.2417	521.6404
2070s	182.9594	186.8206	94.35597	464.13597	497.7451
2090s	230.2719	212.8851	60.11993	503.27693	458.6051

**FIGURE 7:**
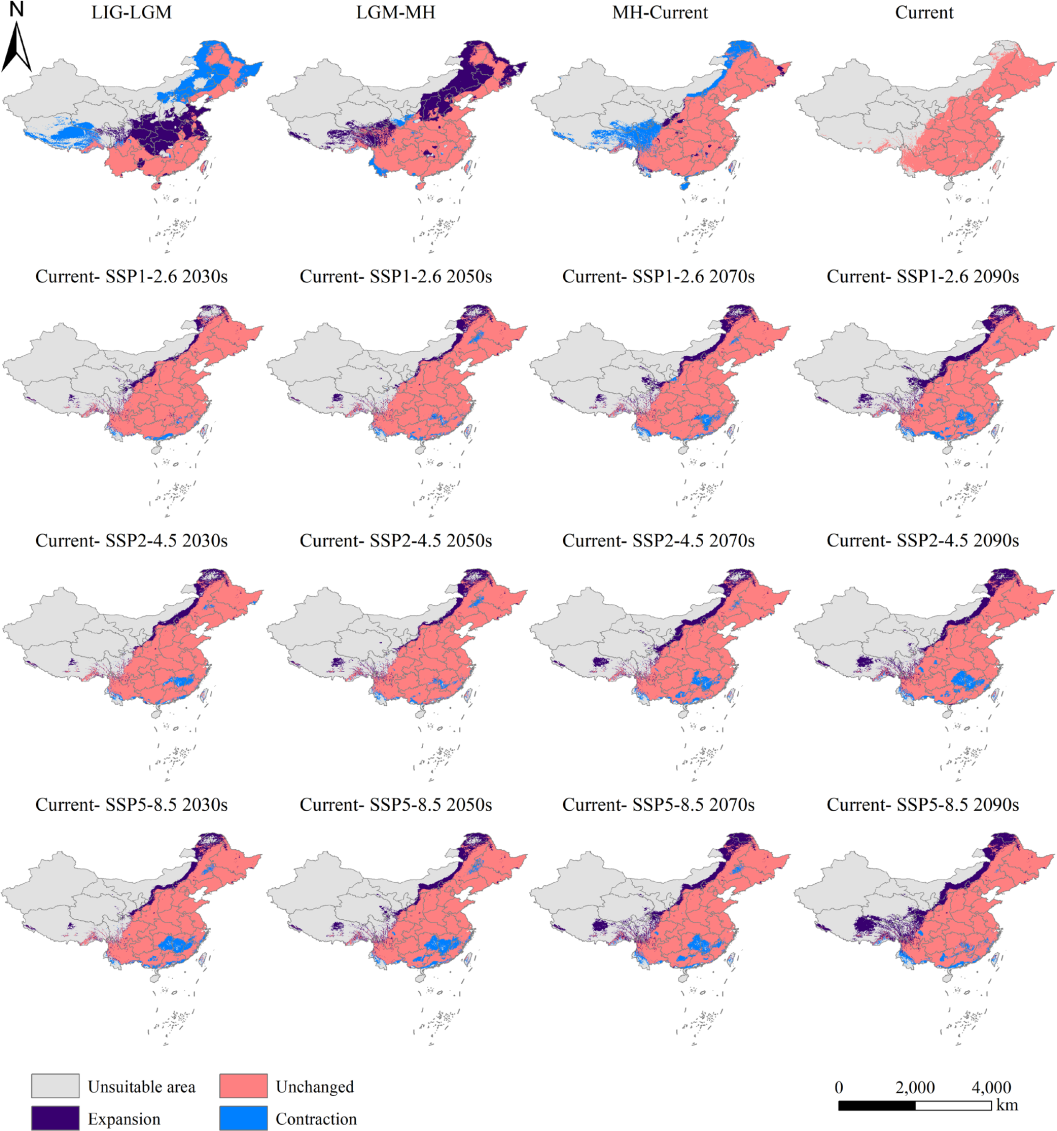
Unchanged, contracted, and expanded suitable habitats for *P. grandiflorus* under different periods and climate scenarios, including last interglacial (LIG), last glacial maximum (LGM), and mid‐holocene (MH).

The area of suitable habitats during the LGM period was close to that during the LIG period. However, these habitats were primarily concentrated in South China (Figure 6). Compared to the LIG period, the extent of highly suitable habitats decreased significantly by about 400,000 km^2^, whereas moderately suitable habitats expanded by about 300,000 km^2^. Figure 7 and Table 4 illustrate the changes in suitable habitats between LIG and LGM, LGM and MH, and MH and the present. Compared to the LIG period, suitable habitats during the LGM expanded in regions such as Hunan, Hubei, Chongqing, and Shanxi while contracting in northeastern China (Figure 7).

**TABLE 4:** Dynamic change of potential suitable habitat for *Platycodon grandiflorum* .

Climate scenario	Area (×10^4^km^2^)				Proportion of area (%)			
	Contraction	Unchanged	Expansion	Total	Contraction	Unchanged	Expansion	Total
LIG‐LGM	152.09	210.11	130.32	−21.77	41.99	58.01	35.98	−6.01
LGM‐MH	16.64	323.79	181.39	164.75	4.88	94.87	53.15	48.27
MH‐current	102.53	402.64	15.17	−87.36	20.30	79.70	3.00	−17.29
Current‐ SSP1‐2.6								
2030s	10.19	408.40	38.36	28.16	2.43	97.57	9.16	6.73
2050s	17.21	401.39	43.02	25.82	4.11	95.89	10.28	6.17
2070s	21.57	397.03	58.63	37.06	5.15	94.85	14.01	8.85
2090s	26.01	392.59	70.00	43.99	6.21	93.79	16.72	10.51
Current‐ SSP2‐4.5								
2030s	19.28	399.32	45.69	26.42	4.61	95.39	10.92	6.31
2050s	15.40	403.20	48.73	33.33	3.68	96.32	11.64	7.96
2070s	24.85	393.75	62.63	37.78	5.94	94.06	14.96	9.03
2090s	30.04	388.56	69.07	39.03	7.18	92.82	16.50	9.32
Current‐ SSP5‐8.5								
2030s	33.42	385.18	46.14	12.72	7.98	92.02	11.02	3.04
2050s	37.10	381.50	58.75	21.65	8.86	91.14	14.03	5.17
2070s	31.17	387.42	76.71	45.54	7.45	92.55	18.33	10.88
2090s	21.77	396.83	106.45	84.68	5.20	94.80	25.43	20.23

During the MH period, the total area of suitable habitats reached 5,051,717.6 km^2^, the largest among all scenario periods. These habitats were distributed across Northern and Southern China. Highly suitable habitats expanded into northeastern Inner Mongolia (Figure 6). Compared to the LGM, lowly and moderately suitable habitats during the MH period increased significantly by 612,073 and 818,935 km^2^, respectively. In contrast, highly suitable habitats rose by only about 200,000 km^2^. Compared to the LGM, suitable habitats during the MH period expanded by 48.27% in regions such as Beijing, Shanxi, and northeastern Inner Mongolia (Figure 7).

### 3.3 Present Prediction

The overall area of suitable habitats for *P. grandiflorus* during the present period was 4,185,964 km^2^. The current distribution range was similar to that during the MH period. Highly suitable habitats were predominantly distributed across northern and southern China (Figure 6). Highly suitable habitat covered 1,394,505 km^2^, primarily in Liaoning, Hebei, Shandong, Hubei, Guizhou, Anhui, and other regions (Figure 6). Compared to the MH period, lowly and moderately suitable habitats in the present time decreased significantly by 942,619 and 900,457 km^2^, respectively. Moderately suitable habitats covered about 1,590,112 km^2^, including Heilongjiang, Jilin, Yunnan, Guangxi, Guangdong, and other regions. The results of our research were in better agreement with the actual distribution of *P. grandiflorus* (Dong et al. 2019). Lowly suitable habitats were about 1,201,347 km^2^, including Jiangxi, Hunan, eastern Heilongjiang, and other regions. Compared to the current time, the suitable habitats during the MH period contracted by 17.29%. This contraction was particularly evident in western Sichuan, southern, and northern parts of Inner Mongolia (Figure 7, Table 4).

### 3.4 Future Prediction

Figure 6 shows the suitable habitats of *P. grandiflorus* under future climate scenarios. Relative to the present, suitable habitats in future periods mainly expanded along the northeastern boundary of the current distribution Range. Besides, the distribution range in Hunan and Jiangxi has shrunk to varying degrees (Figure 7). The overall suitable habitats under the 2030s, 2050s, 2070s, and 2090s scenarios consistently increased. This trend may be attributed to a slight decline in highly suitable habitats and a significant expansion of lowly and moderately suitable habitats. The largest suitable habitat area occurred in the 2090s under the SSP5‐8.5 scenario, reaching 5,032,769.3 km^2^, significantly larger than other scenarios. Lowly and moderately suitable habitats increased dramatically to 2,302,719 and 2,128,851 km^2^, respectively, whereas highly suitable habitats decreased significantly to 601,199.3 km^2^. Compared to the present, the suitable habitat area increased by 846,800 km^2^ (20.23%) (Figure 5 and Table 4). The SSP1‐2.6 and SSP2‐4.5 scenarios showed an approximately 10% increase in the overall suitable habitats compared to the present (Table 4).

### 3.5 Shift in the Distribution Centroid of the Suitable Habitats

**FIGURE 8:**
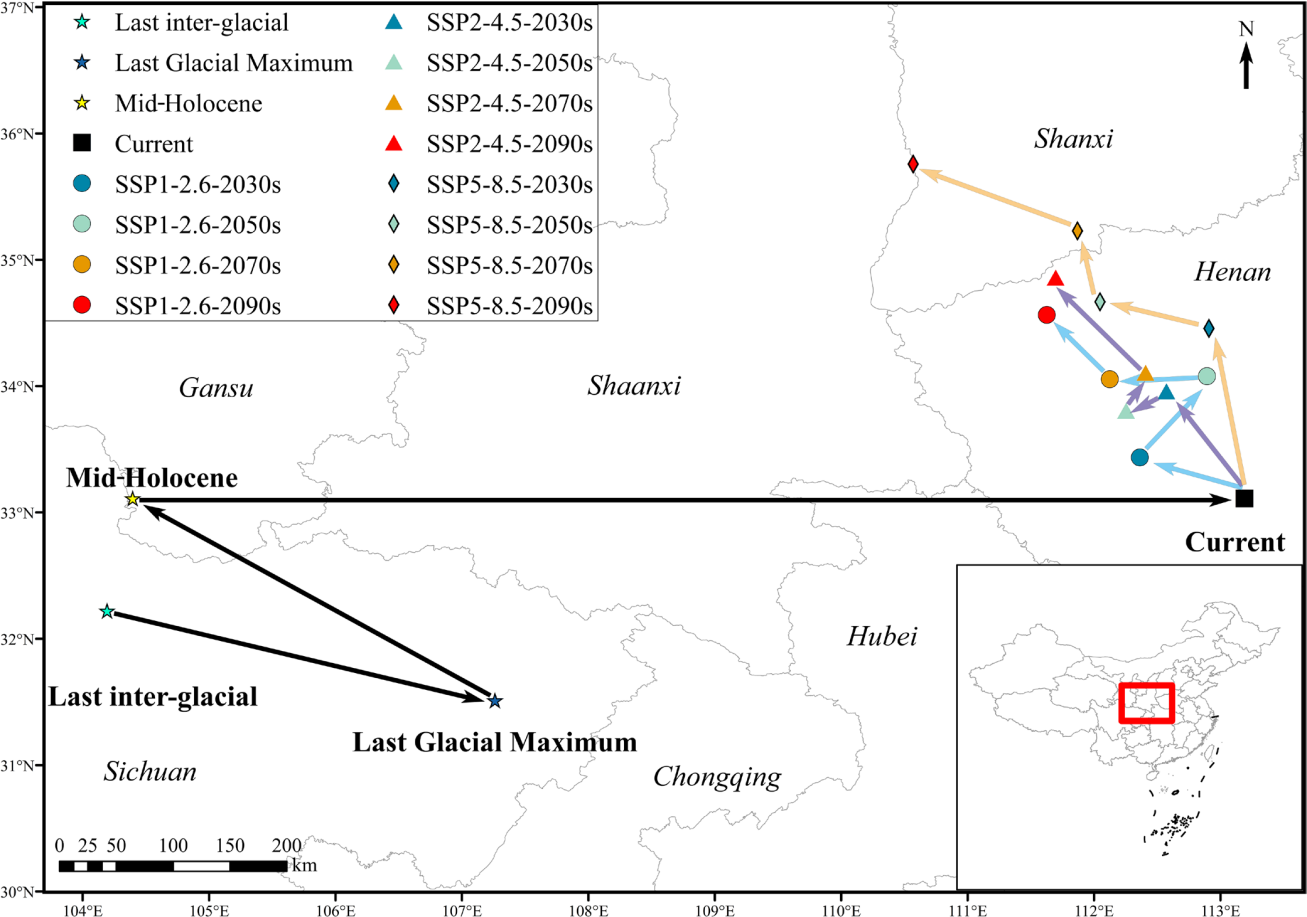
The core distribution shifts under different climate scenarios for *P. grandiflorus* . Arrows display the magnitude and direction of predicted change over time.

The current distribution centroid of *P. grandiflorus* is located at 33°06′39″ N 113°11′25″ E, in Nanyang City, Henan Province (Figure 8). During the LIG, the centroid of suitable habitats was situated in Aba Tibetan and Qiang Autonomous Prefecture, Sichuan Province (32°13′23″ N, 104°11′35″ E). This location is 849.410 km away from the current distribution centroid. During the LGM, the centroid shifted southwest to Bazhong City (31°30′47″ N, 107°15′40″ E), Sichuan Province, 585.675 km away from the current centroid. During the mid‐Holocene, the centroid moved northwest to Longnan, Gansu Province (33°06′46″ N, 104°23′45″ E), 820.592 km away from the current centroid.

**FIGURE 9:**
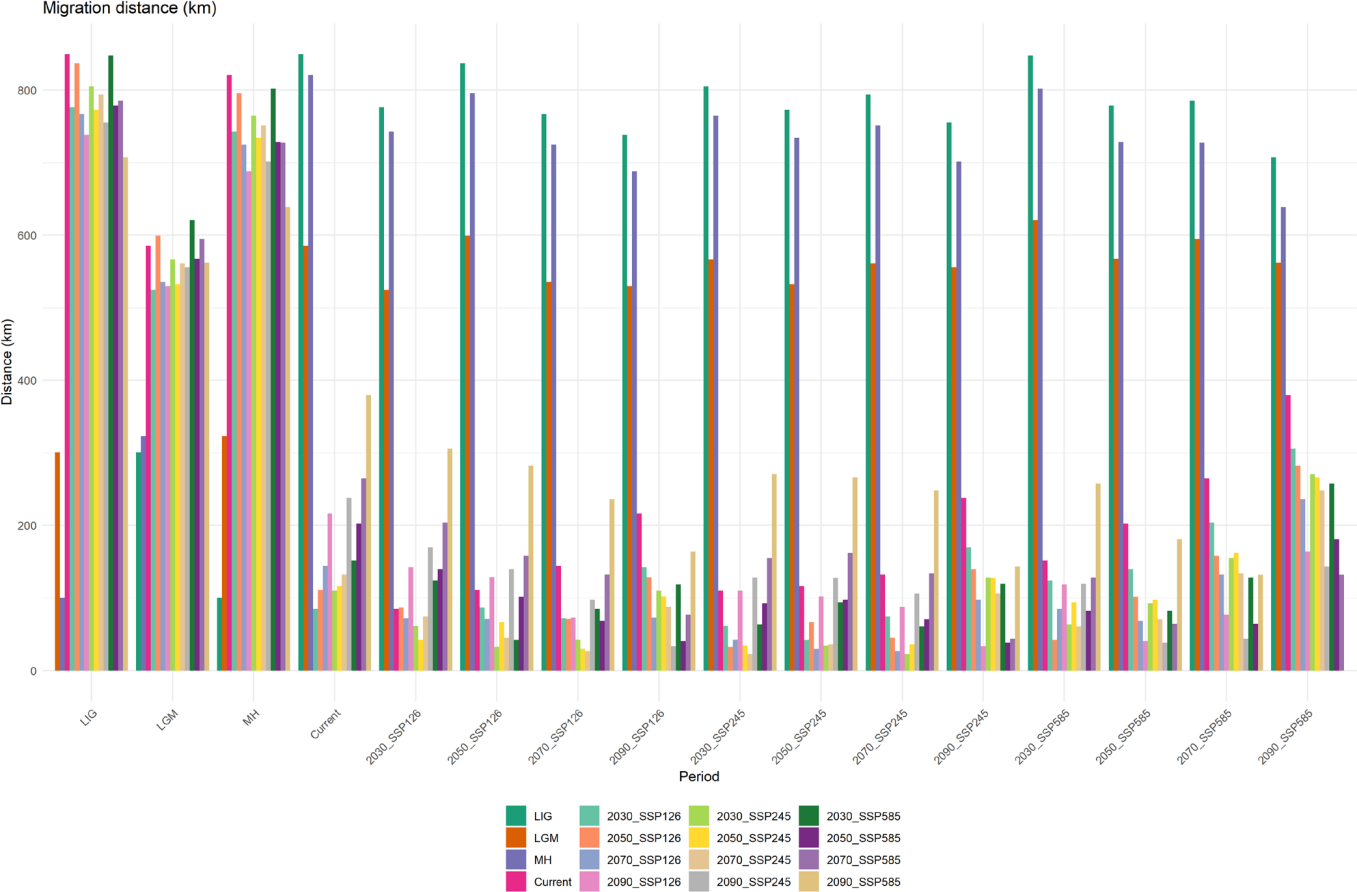
Migration distance of cores for suitable habitats under different climate scenarios.

Under the SSP1‐2.6 scenario, the geographical distribution centroids of *P. grandiflorus* during all periods were nested in Nanyang City (33°26′08″ N 112°21′46″ E), Pingding City (34°04′42″ N 112°53′36″ E), Luoyang City (34°03′16″ N 112°07′29″ E), and Sanmenxia City in Henan Province (34°33′49″ N 111°37′36″ E). Under the SSP2‐4.5 climate scenario, the centroids of habitat areas for all future periods were also distributed in Henan Province (Figure 7). Under the SSP5‐8.5 scenario, the geographical distribution centroids of *P. grandiflorus* during the 2030s, 2050s, 2070s, and 2090s were located in Zhengzhou City (34°27′25″ N 112°54′33″ E) and Luoyang City (34°40′04″ N 112°02′50″ E) in Henan Province, as well as Yuncheng City (35°13′44″ N 111°52′10″ E) and Linfen City (35°45′28″ N 110°34′10″ E) in Shanxi Province. In the 2090s, the distances between the potential future centroids and current centroid were 216.586 km, 238.032 km, and 379.757 km under the SSP1‐2.6, SSP2‐4.5, and SSP5‐8.5 scenarios, respectively. Overall, the distribution centroids during the LIG, LGM, and MH periods are clustered but far from those in the current and future periods (Figure 9). The current distribution centroid was close to those of the future periods.

## 4 Discussion

Only one species of the genus Platycodon is identified in the family Campanulaceae, which is *P. grandiflorus* and mainly distributed in China, Japan, Korea, and some areas of Siberia. It was a typical Chinese medicinal material that was used as food. Due to this high demand, the principal source is cultivated by artificial propagation. Therefore, a quantitative appraisal of the ecological factors affecting *P. grandiflorus* abundance is required. In order to promote the cultivation of *P. grandiflorus* and improve the quality of medicinal materials, it is necessary to understand its potential habitat and conduct a suitable environment zoning study of the whole country. Using MaxEnt‐based ecological niche modeling, we predicted and identified the key bioclimatic variables that explained the geographic distribution of *P. grandiflorus* in China across several past, present, and future climate scenarios based on collection distribution data.

### 4.1 Model Performance

Due to its accuracy and stability, the MaxEnt model has been the most commonly implemented (Yang et al. 2024). Using biased sampling data in the MaxEnt model has shown a tendency for overfitting and false‐positive predictions in a few previous studies (Yackulic et al. 2013; Araújo and Guisan 2006; Kadmon et al. 2004). Next, we defined the sampling points of the distributed data and solved this problem by optimizing all model parameters. The final optimized model provides excellent predictive performance and credible experimental results with an AUC value score of 0.891.

### 4.2 Response of the Geographical Distribution Patterns of *P. grandiflorus* in China to Environmental Change

Reconstruction of the *P. grandiflorus* distributions across ages provides some insights into the historical dispersion and evolutionary fitness of this species. The LIG climate was warm and west (Wu et al. 2006, 2007). The LGM was characterized by extreme conditions, while that of the mid‐Holocene is similar to modern climate (Tarasov et al. 2007; Turner et al. 2010; Zheng et al. 2013). The oscillating movements of glacial and interglacial periods profoundly influenced the distributional ranges and gene flow of the extant flora (Hewitt 2004). This study illuminates how *P. grandiflorus* has responded to climate change since the ice ages.

During the LIG period, *P. grandiflorus* was distributed widely in two localities (northeastern and southern China), different from the present. These LIG years and subsequent temperature gradients might be less important to *P. grandiflorus* ; according to our data, they persisted in warm and damp environments throughout this period. Since the last glacial maximum (LGM), it has been distributed from northeastern to central and southern China. Our results show that temperate plant taxa, which are known to have a high degree of ecological adaptability, have retreated to lower latitudes during the LGM period (Zhao et al. 2021). The displacement of vegetation zones southwards was substantial during the LGM. This shift may have been imposed by an expansion of the deserts and steppes in northwestern China compared with the present day (Wang and Sun 1994). Moreover, the LGM climate was overall significantly drier than the modern climate. The temperature, solar global radiation, and other conditions were more suitable for the growth and development of P. *grandiflorus* in South China. During the LGM, significant climate changes recast the community assemblage, but some species experienced rapid range changes (Jiang et al. 2019; Pellissier et al. 2016). The LGM climate had a more substantial effect on the total and the highly suitable habitat of *P. grandiflorus* than of the other periods studied, with the areas of habitat the least examined.

The distribution area of *P. grandiflorus* showed a marked increase during the mid‐Holocene (MH) period (occupying almost half of China compared to the LGM period), suggesting that the distribution range was substantially impacted by climate during the MH period. During this period, the overall suitable habitat of *P. grandiflorus* was more widely distributed than in other historical periods. This expansion may track periods of optimal habitats when East Asian hydrological settings (8.6–4.4 ka) were relatively dry during other Holocene periods (Li and Pritchard 2009; Tian and Jiang 2015). This analysis showed that the middle Holocene represented the period when *P. grandiflorus* habitat was most suitable. Meanwhile, the suitability of the available habitats expanded for some residual plant groups that survived in the MH model agreed with the other plant group research (Bai and Zhang 2014; Zheng et al. 2013; Zhao et al. 2021).

The current potential distribution of *P. grandiflorus* covers most regions of China, except for the northwest. The predicted outcomes align with the known distribution of *P. grandiflorus* in China (Dong et al. 2019). Our results demonstrated that future expansions in geographic distribution are primarily concentrated along the northwestern boundaries of the present suitable habitats. The boundaries of suitable habitats are sensitive regions where species respond to global environmental changes (Diamond et al. 2011; Thuiller et al. 2005; Zhao et al. 2021). Additionally, contractions of suitable habitats are observed in parts of Hunan, Jiangxi, southern Yunnan, and the Guangxi Zhuang Autonomous Region, likely due to future local climate changes. Overall, suitable habitats increased under all future SSP scenarios relative to the present. The SSP5‐8.5 scenario in the 2090s led to the most significant expansion, with an increase of 503.27693 km^2^. The increasing tendency in suitable habitats for *P. grandiflorus* is compatible with studies on other plants, such as *Choerospondias axillaris* (Ye et al. 2019) and *Ziziphus jujuba* (Zhao et al. 2021). However, it differed from findings for species like *Polyporus umbellatus* (Guo et al. 2019), *Quercus lamellosa* (Guo et al. 2021), and *Taxus* (Wu et al. 2022). However, contractions of highly suitable habitats are observed in all future scenarios compared to the present. The greenhouse effect and global warming will intensify as greenhouse gas emissions continue to rise. China's climate is projected to become warmer and wetter (Yan et al. 2021), potentially impacting the highly suitable habitats of *P. grandiflorus* .

In our study, the centroid of *P. grandiflorus* during the mid‐Holocene period shifted significantly eastward compared with the present period. This shift might be related to the distribution of artificial *P. grandiflorus* planting areas. Under the SSP1‐2.6‐2030s scenario, *P. grandiflorus* exhibited the shortest migratory distance, with a geographical shift of approximately 85.093 km from the present centroid. Under the SSP5‐8.5‐2090s scenario, *P. grandiflorus* migrated northward by 379.757 km from the present centroid, representing the farthest migration among all future scenarios. With future global warming, the suitable habitats of *P. grandiflorus* are projected to shift toward higher latitudes. This pattern holds for the 2030s, 2050s, 2070s, and 2090s decadal time slices. Global warming migrates many species to higher latitudes (*Cyananthus*, He et al. 2019; *Quercus lamellosa*, Guo et al. 2021; *Taxus mairei*, Wu et al. 2022). Species can range in latitude or elevation in response to global climate changes (Davis and Shaw 2001).

### 4.3 Prediction of the Relationship Between Environmental Factors and the Distribution Shifts of *P. grandiflorus*

Throughout its life cycle, including vegetative growth, development, reproduction, and genetic adaptation, *P. grandiflorus* is impacted by its living environment (Ma and Sun 2018; Fang et al. 2024). Influencing factors of its distribution are found in our results, including precipitation‐related variables (bio13, prec07, and prec09), which reflected the moisture preference of this species (Moon and Yoon 2018; Nguyen et al. 2022). Among these, the precipitation of the wettest month (bio13) is the most significant factor for the survival of *P. grandiflorus* . Besides, our results demonstrated that *P. grandiflorus* predominantly thrives in the Temperate and Subtropical Monsoon Climate, where mild and humid summer conditions favor its growth.

Although precipitation is the primary factor influencing the potential geographic distribution of *P. grandiflorus* , temperature fluctuations also play a significant role. The optimal July minimum temperature (tmin07) for *P. grandiflorus* ranges from 19.7°C–25.3°C, indicating that this species thrives in moderate temperatures. Studies on the distribution patterns of endemic plant groups have shown that many relict plants prefer warm climatic conditions (López‐Pujol et al. 2011). Our findings are consistent with these observations. In addition, temperature fluctuations significantly impact the latitudinal migration of plants under climate change. As the global greenhouse effect intensifies, heat‐limited areas are predicted to expand, leading to shifts in plant growth zones (assuming no feedback from precipitation factors). However, this may pose challenges for populations in hotter climates. Habitats in southern regions are probably less suitable, leading to northward migration of *P. grandiflorus* populations. This aligns with our inferred migration patterns.

### 4.4 Conservation and Cultivation Management

The root of *P. grandiflorum* is one of the central medicinal parts and has been utilized as a traditional medicine and food ingredient in Asia (Lee et al. 2014; Kim et al. 2016; Kwon et al. 2017). Studies showed the influence of climatic factors (such as temperature and precipitation) on rooting (Fang et al. 2017; Zhou et al. 2019; Reich et al. 2018). Studies have shown that excessive precipitation can allow for rotten roots. Therefore, improving land drainage conditions should also be emphasized (Dong et al. 2019). Furthermore, global warming is expected to alter the amount, distribution, frequency, and intensity of precipitation (Myhre et al. 2018). Inappropriate water and heat conditions are not conducive to seed rooting, leading to a decline in population numbers. So, we should predict the species showing relatively good development at the ideal temperature (between 19.7°C and 25.3°C) according to its present habitat requirements; it was also indicated in another study that the roots and stems of the plants are susceptible to alterations in the annual precipitation and temperatures (Li, Bi, et al. 2016; Wei et al. 2015). These factors are related to photosynthesis pathways and water absorption (Austin 2002; Low et al. 2021).

After analyzing the climatic data, it is inferred that *P. grandiflorus* will migrate to higher latitudes. Apart from the directional effects of habitat alterations on the evolving conditions of plant growth, habitat changes may also impact socioeconomic aspects. A loss of livelihood from reduced access to resources is expected due to potential habitat shifts, which could result in lower availability of plants collected for household food and market sales (Williams et al. 2021). Thus, integrating mixed planting with other agricultural or non‐agricultural activities could motivate communities to reach diversified income sources (Delgado and Siamwalla 2018). Medicinal plants are raw materials for pharmaceuticals, herbal supplements, and cosmetics, and thus, habitat shifts may also affect the workings of both the pharmaceutical and herbal industries. In addition, habitat shifts may contribute to further disruptions in supply chains, raise costs, and decrease the availability of key ingredients (Applequist et al. 2020; Alum 2024). Therefore, we must enhance the cultivation of these plants to maintain a steady supply for industries (Hishe et al. 2016; Dong 2021). Finally, habitat shifts will increase conservation and restoration costs, such as habitat restoration (Kimball et al. 2015) and ex situ conservation (Li and Pritchard 2009). Incentives for conservation practices can be financially incentivized for farmers and communities (e.g., subsidies and grants; Garbach et al. 2012).

Except for the SSP5‐8.5_2090 among all future periods, the total suitable habitats of *P. grandiflorus* will increase slightly. However, climate change might risk the highly suitable habitats for this vital medical herb. This requires region‐specific guidelines and conservation strategies to be developed for policymakers, farmers, and conservationists. Policymakers may design legislation and regulations that influence protection (Löbl et al. 2023), including but not limited to the governance and management of nature reserves to promote in situ conservation within natural habitats (Wang and Li 2021) and the allocation of financial contributions for conservation (McKinley et al. 2017; Anthony 2024). One possible solution is for farmers to practice sustainable agriculture. These might involve agro‐forestry (Wilson and Lovell 2016; Wezel et al. 2014), organic farming (Soni et al. 2022; Gamage et al. 2023), and training programs. Conservationists must assess conservation status and monitor the population size of multiple species (Lindenmayer and Likens 2010). This, in turn, is increasingly important for creating awareness and education of the public on the possible impacts of human behavior on plants using promotional activities and mass media (Jacobson et al. 2015).

## 5 Conclusions

Our study showed that temperature and precipitation significantly affected the suitable habitats of *P. grandiflorus* . Our research can deepen our understanding of the impact of paleoclimate, present, and future climate on the dynamic distribution of species in China. The habitat of the mid‐Holocene period was the most suitable habitat for *P. grandiflorus* . The suitability of habitats is likely to advance to higher altitudes or latitudes under warmer future climate conditions. Such changes can affect plant species supplying medicinal products and will directly affect the availability and quality of those species by altering habitat structures and species compositions (Parry 2007). This expected change in distribution highlights the need for adaptive management plans to guarantee the long‐term viability and sustainability of the *P. grandiflorus* population. In addition, climate change will likely reduce areas of highly suitable habitat and demand urgent and targeted interventions. As global warming continues, the findings of this study provide a strong theoretical foundation for formulating effective strategies to enhance the resistance of *P. grandiflorus* . Predicting species distribution ranges is crucial for informing rational cultivation and conservation strategies. These efforts will guarantee the delivery of sustainable pharmaceutical resources and quality to the forthcoming generations.

## Author Contributions

**Chun‐Jiao Li:** conceptualization (lead), data curation (lead), formal analysis (equal), funding acquisition (lead), investigation (lead), writing – original draft (lead), writing – review and editing (lead). **Xin‐Tong Xie:** conceptualization (supporting), data curation (supporting), formal analysis (supporting), methodology (supporting), resources (equal), software (equal). **Tuo Shi:** conceptualization (supporting), validation (supporting), visualization (supporting), writing – review and editing (supporting).

## Conflicts of Interest

The authors declare no conflicts of interest.

## Supporting information


**Figure S1.** Multicollinearity test by using Pearson correlation coefficients of all environmental variables.

## Data Availability

We used open‐access data from GBIF (Global Biodiversity Information Facility, https://www.gbif.org/), CVH (Chinese Virtual Herbarium, http://www.cvh.ac.cn), NSII (National Specimen Information Infrastructure, http://www.nsii.org.cn), and WorldClim (http://www.worldclim.org/). The distribution records of *P. grandiflorus* have been uploaded to the public database through Figshare (DOI:10.6084/m9.figshare.28521665).
