# Supplementary material for: Distribution Patterns of Platycodon grandiflorus From the Last Interglacial Period to the Future by Ecological Niche Modeling
Source: Ecol Evol. 2025 Mar 30;15(4):e71198. doi: 10.1002/ece3.71198 (PMC11955282; doi:10.1002/ece3.71198)
Supplement: Supplementary file 1 — Figure S1. Multicollinearity test by using Pearson correlation coefficients of all environmental variables. [file ECE3-15-e71198-s001.docx]

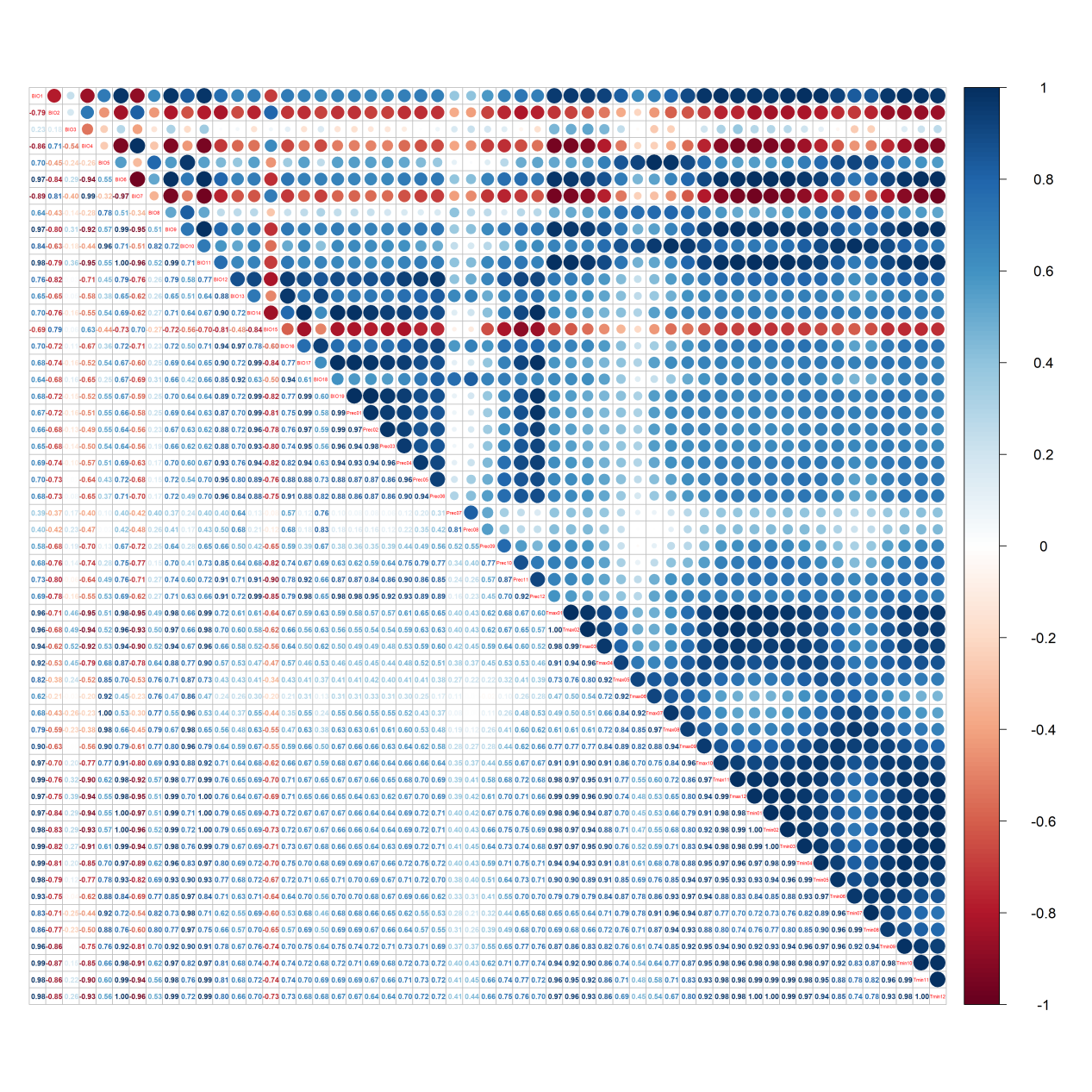


**Figure S1** Multicollinearity test by using Pearson correlation coefficients of all environmental variables.
